# British Medical Association

**Published:** 1872-09-01

**Authors:** 


### BRITISH MEDICAL ASSOCIATION.

We quote the following regarding the proceedings of the annual meeting of the British Medical Association, held at Birmingham last month, from *The Doctor*, Sept. 1st, 1872:

The President, Mr. Alfred Baker, Senior Surgeon to the Birmingham General Hospital, delivered an eloquent and interesting address, welcoming the visitors to Birmingham.

The address on surgery was delivered by Mr. Oliver Pemberton, Surgeon to the General Hospital, and Professor of Surgery in Queen's College, Birmingham. The first part of it he devoted to some points connected with the treatment of aneurism. He said:

Professor Lister's improvement in the Hunterian operation, by which the permanent closure of the artery at the spot tied can be insured, without dividing the coats of the vessel, at once effects a complete change in some of the most important conclusions that for long years have guided us in our treatment of aneurism. One of the greatest dangers attending the Hunterian operation has hitherto been considered to be the application of the ligature immediately beyond any considerable branch of an artery. This impression has deterred surgeons from applying a ligature to that portion of the artery which otherwise would have seemed to them best adapted for the purpose. That an abiding coagulum will form under certain circumstances in the vicinity of almost any number of branches on the proximal side of a ligature, I am perfectly satisfied; but the attainment of this success in many cases depends on a fact which it is almost impossible for the surgeon to estimate beforehand; that is, the facility with which the blood will coagulate or deposit its fibrin in any particular instance.

. . . . . . . . . . .

Apart from this question of coagulation, I feel warranted in expressing my conviction that too much stress has been laid on the disturbing influence of a large branch or branches taking origin close to the part of the vessel tied. If, however, we are to believe the teaching of Professor Lister ("Observations on Ligature of Arteries." Edinburgh: 1869), it will be of little moment in future whether a plug form on either the proximal or distal side of the ligature at all, so long as the "prepared catgut" insures permanent closure of the vessel at the spot tied, without severance of the coats, and, consequently, without liability to secondary hemorrhage.

I am glad, before such a meeting, to be able to express my unbounded admiration of, and confidence in, the use of the animal ligature, as placed before us by Professor Lister. If the so-called "antiseptic system" has effected no more for surgery than to give us the means of effectually closing an artery without cutting it through, and without suppuration, it has in this placed the crowning glory on the treatment of aneurism, for which it has waited since the time of Hunter.

I shall now endeavor to show that the principles of treatment in the methods of flexion, compression of the sac, and manipulation, are one and the same.

The method of flexion can only be applicable to certain arteries. All that it is needful to do is to keep the limb flexed, not continuously, but to such an extent as to alter the relations between the orifices of ingress and egress, and the fibrinous laminæ of the sac. Some of these laminæ become, as it were, dislocated; and protrude more or less into the stream when a fresh deposit of fibrin occurs, and so the cure is gradually effected.

The exercise of pressure on the artery above the angle of the flexion appears to me useless. What we want is a stream of blood flowing into the aneurism, that it should be more or less retarded there, and that there should be present something in the nature of a foreign body—for example, the fibrous laminæ, on which blood would coagulate and deposit its fibrin. This retardation of the blood in the sac can be effected by a gentle compression of the artery on the distal side of the aneurism, as I strongly hold that what we want in these cases is a deposition of fibrin rather than a coagulation of blood. For, surely, the slow deposition, layer after layer, of solid fibrin in the sac until the filling-in is complete, is a surer guarantee against subsequent mishaps than if it were closed by a mass of suddenly coagulated blood.

I entertain the opinion that the compression of the sac ought to be used more frequently than it is now. The principle of this proceeding is exactly the same as flexion; we want simply to alter the relations of the laminated fibrin to the cavity of the aneurism, so as to bring about a further deposition of fibrin on the projecting surfaces of any of the displaced laminæ. The pressure need not be continuous. It should be very gentle. It need not, even, be distributed uniformly. But it must ever be borne in mind that if it be carried to such an extent as to empty the sac, and to press one wall against the other, then a cure cannot occur. The very conditions under which a cure is possible are here ignored. Blood must pass through the sac. It must not pass through too rapidly; and I now think that this would be facilitated by gentle pressure being made on the artery below the aneurism.

Reduce the force and volume of the blood current by any carefully considered measures, and we follow out the reasoning of Brasdor and Wardrop, in the distal ligature; a reasoning which is rendering amenable to the treatment of internal aneurisms hitherto beyond surgery; a reasoning that has the authority of nature's own proceedings to recommend it, from the fact that it is more or less identical with the mode in which the so-called spontaneous cures are brought about.

I cannot but regard the treatment by manipulation to be based on exactly similar principles to those on which the methods I have just alluded to are founded. No forcible pressure to detach fibrinous laminæ, in my judgment, ought to be used; as the result would be the almost certain separation of small portions of the clots, which would be carried into circulation, and would eventually plug the smaller vessels, causing symptoms according to the functions of the parts which the plugged vessels supply. For I must own I have not been able to see how these clots could be located at either outlet, to be fixed by arrangement, as it were, at a spot where it is simply impossible to be assured that they could effect a lodgment. All that is necessary is that the aneurism should be gently manipulated, so that the laminæ of fibrin in its interior should occupy a different position to that which they had previously held with reference to the two orifices of the sac; and in order that the blood should not be allowed to pass out of the sac too freely, if I have an opportunity, I shall endeavor to compress the distal artery in accordance with the principles I have been advocating.

I have now to call your attention to what I believe to be a not uncommon result of the cure of aneurism, after it has been effected for some time; I mean the formation of varicose aneurism, or aneurismal varix. I shall first relate two cases. In 1844, my late colleague, Mr. Amphett, tied the superficial femoral for an aneurism of the artery as it enters Hunter's canal. The patient was 41, and a soldier. There was nothing unusual at the operation, and the ligature was thrown off on the nineteenth day. Ten days subsequently, there was arterial hemorrhage from the seat of the ligature. This recurred in ten days, and a third time in fourteen. Pressure on the arch was used, and the patient recovered. He remained well for upwards of three years, when a tumor formed at the seat of operation, which was evidently an arterio-venous aneurism. With this coming under the care of my colleague, Mr. Baker (our

President), he died with a drunken pleurisy, just five years from the date of the operation. I was fortunate in being able to dissect his vessels. The femoral artery had formed an aneurism at the seat of the operation as large as a hen's egg, and the femoral vein communicated with the artery by a large opening. The former aneurism was cured, and the artery between it and the seat of the ligature was impervious.

LITHOTOMY.

Mr. Pemberton next considered the subject of Lithotomy. Advocating the median operation, he said:

I shall be prepared for it to be said of my advocacy of median lithotomy, "The statistics of your own cases are against you." My answer is, "Statistics are not everything." A case may end just as well one way as another, though the troubles on the journey differ widely, and no one will question that lateral lithotomy in children is eminently successful. But every operator who has sufficiently tried any given two methods of procedure, has a right to say which of the two he prefers; and therefore it is that I say, when I reflect on the anxiety that I endured in watching the threatenings of mischief in children cut by the lateral operation, I rejoice that I have cause for it no longer, notwithstanding the general good fortune that attended my practice with that method.

And now as to the cases where the median operation should not be selected. In any instances where the finger is not likely to reach the bladder, so that instrumental dilation would be required, the lateral operation should be preferred. The reason I use my finger is because I have more control over it than over an instrument. I can regulate the one, not the other. I would sooner cut than lacerate at any time, and I consider that the use of instrumental dilation in this operation means laceration. You may use it, on and off, with impunity, but it is a most destructive instrument—reviving all the dangers of the discarded Marian. I attribute the peritonitis, which carried off my single fatal case, solely to the laceration of the neck of the bladder that of necessity followed its use. I repeat, the only dilator must be the finger, and so long as the neck of the bladder can be widened by this sufficiently to allow of the removal of a stone without laceration, I shall deem it a part of my duty to advocate the adoption of this form of median lithotomy.

I hope, however, my observations will not be misunderstood. I am second to none in admiring what Cheselden practiced, and what Liston and Furgusson have brought to perfection—the lateral operation for stone. I have been surrounded during the whole of my professional life by teachers and colleagues who have had unusual opportunities for practice, and who have realized brilliant successes in this very operation; but, in my opinion, it is not the most desirable operation to perform for all stones, at any age and under any circumstances, as some would have us believe.

STRICTURE OF THE URETHRA.

Mr. P. then proceeded to speak about stricture of the urethra: It is to me remarkable, but it is true, that the views entertained by the highest surgical authorities of the day differ on no subject so widely as on the particular system they adopt and recommend in the treatment of stricture. Simple dilatation and rest, I am thankful to say, have had a great following, and, if I mistake not, will yet rise into higher position. The main quarrel is between the advocates of internal as opposed to external division. The late Professor Syme (*Stricture of the Urethra*, p. 21, 1855) thought he had effectually put an end to the use of those "dreadful engines," as he termed M. Reybard's instruments; but he was mistaken, for strictures of this day are both cut, split, and torn; and new engines for the purpose multiply, as if the great surgeon had never lived to speak of plunges in the dark with caustic, or of ripping open the urethra by internal section.

Stricture may fairly be defined to be a diminution of the normal diameter of any portion of the urethral canal; and as it must be admitted that the existence of any stricture, however slight, from whatever cause proceeding, and of whatever nature, may sooner or later give rise to serious consequences in the condition of either the bladder or kidneys, it is needful for the surgeon to discover it and cure it as soon as possible. But the real question is in reference to this word cure. Have we to deal with a simple stricture that has resulted from inflammation of the lining membrane of the urethral canal, or with a stricture originally of this kind, which has been aggravated and increased in extent by ill-considered surgical proceedings?

For the first there is a cure by simple dilatation. For the second there properly is no cure. Once organic stricture, always organic stricture, is my belief. Whenever the lining membrane of the urethra has been injured, whether by accident, disease, or by bad surgery, the spot will contract and establish permanent stricture, and I do not believe that the materials constituting such cicatricial narrowing are ever absorbed.

If you endeavor to restore the normal caliber of the urethra under these conditions by ever so well considered a system of dilatation, my opinion is that the contraction will return sooner or later with increased vigor, the natural elasticity of the canal being gone; in other words, dilatation will not effect a cure, and never does effect a cure.

But dilatation, if it be well and properly carried out, will protect the patient against the occurrence of those diseases which, dependent on individual health and mode of life, arise either rapidly or slowly in all cases of stricture. The degree to which it is necessary to carry this may fairly allow of discussion; for I have ever before my mind the conviction that the very means made use of to effect the so-called cure, may become the certain cause of the continuance, and, in many cases, of the increase of the malady.

I think it will be admitted that the tendency to narrowing in cases of stricture differs very markedly in individuals. Some may show few signs of change during many years, others, especially those arising from the effects of laceration by direct violence, certainly, surely, and often rapidly increase. In all cases, treatment by dilatation is necessary; but I doubt myself whether it is needful always to endeavor to restore the standard of the canal to the utmost of its original extent. I believe that there are many cases which admit of being maintained at a standard short of this, depending, however, on the facility with which the contraction yields, and its rate of increase subsequently. And it must never be forgotten that when once this treatment by dilatation has been commenced—no matter how carefully or how thoroughly it may have been done—it will have to be continued, whether at the hands of the surgeon or of the patient, more or less during life.

For my own part, time being given, I do not believe that there is any stricture through which an instrument cannot be passed by a skillful surgeon. This being so, treatment by gradual dilatation follows; and, in my judgment, this should be by the silver catheter, as the safest, simplest, and most certain instrument in the greatest number of hands yet given to us, *bougie a baule* and *bougie alivaire* notwithstanding. If the induration be cartilaginous non-dilatable, or if there be fistula, the treatment by external division on a grooved staff should be adopted as speedily as possible.

Entertaining this view of the permanence of the changes established in the urethra by injury or disease, I am not very likely to favor any internal severance of the lining of the canal, whether by Mr. Holt's method of so-called "splitting," or by any form of internal cutting. I believe a wound is produced just as much in the one case as in the other. I regard those methods as artificially inducing the very conditions which I lament should result from almost unavoidable causes; and I further believe that a shut-up wound on the internal face of the lining of the urethra, is attended by dangers, from which an open wound on the outside face is comparatively free (*a*). I have had occasion to divide the urethra after Professor Syme's method in upwards of thirty cases. In one case only was there a fatal ending, and this from pyæmia. In no case was there a relapse, provided that an instrument was passed from time to time, the frequency of this being determined by individual tendency to re-contraction, once a month to once in three months being about the average; and by this means the caliber of the urethra was without difficulty maintained at its original standard. All the cases that I have seen, save one, have required this continued resort to dilatation, and will require it, in my judgment, more or less during life. For there is no more a cure by this than by dilatation or splitting. In the case that did not require it a fistula remained permanently in the perinæum, letting through a little urine, the general stream flowing by the urethra, which at the end of twelve years shows no disposition to contract.

If the induration of the urethra, and narrowing, be of such an extent as to preclude the idea of dealing with it by external division, I prefer to tap the bladder by the rectum. I do not feel inclined, at present, to divide from the bulb to the meatus; and this literally must be the length of an incision in many of these long-standing cases, if the entire disease is to be dealt with.

There are numbers of these inveterate cases wholly unsuited to external division; but they are eminently calculated to be dealt with by a method which deviates the course of the urine to another channel, in order that rest may heal the fistula, and absorb much of that adventitious material blocking up the natural urethra, which can then readily be found, and have a standard established almost without resort to dilatation.

I frankly say that I do not believe that either internal or external division of any urethra will cause the healing of fistulæ in the groin, buttock, and perinæum, where a man passes his urine, as it has been graphically described, like a watering pot.

Surely, relief by the rectum will stand

(*a*) I will, with Sir H. Thompson, admit its use in narrowings at the external meatus.—*Pathology and Treatment of Stricture, third edition.*

comparison with all the maneuvers that have been suggested from the days of Hunter to Grainger, and from Grainger, who, by the by, belonged to us here, to Gouley and Wheelhouse. I cannot conceive why a patient is to sustain—sometimes for hours together—the distress belonging to hopeless attempts made to trace, in that stage of the disease, an impracticable canal, when the chief cause of the malady—the flow of urine—can be reached and diverted in a moment. Since Mr. Cook published his views (*Medico-Chirurgical Transactions*, Vol. XXXV., p. 153), now just twenty years ago, I have had many opportunities of seeing the results of this proceeding.

I am able confidently to state that it is wholly free from danger. Indeed, I can scarcely conceive death following as a direct result of the operation. So little fear of the proceeding had one of my patients that he has been tapped at least six times for the relief of fleeting attacks of retention, dependent on a rapidly distended bladder, unable to empty itself in the presence of long-standing organic stricture. I have seen him almost within a day or two afterwards as if nothing had occurred. Further no fistula remains, for the opening in the rectum invariably closes after a few weeks.

I have left in the silver canula for three weeks, and have not found inconvenience from its presence; indeed, it appears to me that one of the greatest arguments in favor of its adoption exists in the fact of the position of the canula, which, whilst certainly securing the emptying of the bladder, is wholly removed from the urethra. I am strongly myself of opinion that many urinary cases terminate fatally from urethral irritation, set going and kept up by an instrument retained in the canal in its length.

Some persons are very tolerant of tied-in catheters, whilst others, dependent on a certain idiosyncrasy, cannot sustain with impunity the simple introduction of an instrument. I saw a case in a young man which all but ended fatally from epileptic convulsions, induced by a first catheter; whilst the single introduction of a lithotrite in a man of 77 to measure a large smooth stone that had been carried with impunity for years, set up such an attack of cystitis that death ensued. I was very much impressed by a case in which a man, suffering from complete paralysis from the bladder downwards, owing to concussion of the spine, had a silver catheter tied in his bladder. He appeared sinking fast, and the most profound irritation of the bladder was established. I directed the urine to be drawn off every eight hours, and he began from that moment to amend, and ultimately recovered. Here, doubtless, the true explanation lay not in idiosyncrasy, but in the fact of the existence of disease from the injury. You may leave an instrument in the bladder for years from the perinæum, but you cannot do this with impunity and traverse the length of the urethra. Morbid sympathies become excited in connection with the urethra, which are not produced by the introduction of instruments into other mucous channels.

In what I have said, I have urged the adoption of tapping by the rectum, as affording assured relief to the most inveterate forms of stricture. And in considering the treatment of this disease, I have hitherto limited my observations to cases of stricture of the urethra *per se*, not to those complicated by retention of urine. I must equally urge it, however, as the remedy most reasonable for almost every form of retention. It is the absolute cure of spasmodic stricture; and if, in any given case arising from this cause, after one good effort has been made to obtain relief by ordinary means, there is no success, it should be carried into effect. If retention be present with an impermeable urethra from organic stricture, a double necessity supports its selection, whilst I have yet to learn that it is inadmissible in the retention of old people from enlarged prostate. I know that it can be accomplished in these cases, but of course not so readily as if the rectum had only its ordinary contents; and I am quite satisfied that far less irritation would be produced in the majority of these diseases, where death so often directly results from the effects of instrumental measures, by the presence, at the most depending part of the bladder, of a harmless tube, calculated to secure the removal of all urine secreted, and thus master that inevitable decomposition which is not overcome by any other method in use, for the simple reason that one and all fail to empty the bladder. If the membranous urethra bulge behind a stricture, or if an abscess opened in the perinæum suggest a ready path to the bladder, by all means let a female catheter effect, through the perinæum, what otherwise, I maintain, can be accomplished by the rectum.

Some years ago I asked the question, "Can the urethral canal be permanently restored whenever any complete and considerable portion of its length has been entirely destroyed?" I believe the answer must yet be "No." I had then a boy of sixteen, with at least two inches completely destroyed by burning; and, believing this, I established him with a silver perinæal tube, through which he now (aged 27) passes his urine without trouble ; but there is nothing in the growth of the parts that tempts me to interfere, for I know the whole circle of the canal must be gone.

I think, however, that if only a streak of mucous membrane lingers about the part, an efficient connection can be re-established even after the lapse of many years.

Dr. Evory Kennedy, late Master of the Dublin Lying-in Hospital, delivered the Address at the Opening of the Section on Midwifery, in which he related the following cases:—

Case II. *Excision of part of neck of Uterus.*—Dr. Kitson, of ——, brought a patient from the country, suffering from ulceration of the os uteri. The neck was enlarged considerably, and elongated, the ulcer, which impressed us both as presenting all the characters of malignancy, occupied about one-third of the neck. It had taken a rapid course, bled at intervals freely, and upon the slightest touch, and was attended with pain, sleeplessness, and marked constitutional disturbance. It was, however, circumscribed and limited to the part ulcerated; the remainder of the neck and os being healthy to the appearance and touch, although larger than natural. The lady had borne children. The part of the neck engaged extended from the posterior along the left side of the os, and the diseased structure appeared to occupy the entire substance of the wall. Under these circumstances the case promised little or nothing from the application of the ordinary caustics, and the choice appeared to lie between the free application of potassa fusa and excision. The latter was determined on ; first, because of the limited extent of the part engaged; secondly, because of the apparent malignancy; thirdly, from the difficulty of destroying by the potassa the whole diseased structure, without extending its action to the adjoining vital parts. On the other hand, the diseased structure came well within our view ; the neck was long, affording facilities for the use of the knife. The patient was placed on her back. The vaginal wall and labia were distended by my four brass tractors, firmly held by Dr. Hans Irvine, and Dr. Kitson. An ebony spatula, nine inches long, and half an inch broad, was introduced and placed within the os. This I held firmly in my left hand, whilst I introduced the scalpel which I now exhibit, which, you perceive has a handle seven inches long, while the blade is scimitar-shaped. Cutting from without inwards towards the resisting spatula, commencing near the point of junction with the neck and body of the uterus, above the central part of the diseased structure, by two divaricating incisions Λ, a triangular section was removed. I was prepared to draw the uterus down with the double tenaculum; but this was unnecessary, from the perfect manner in which my assistants used their tractors. This allowed me the assistance and security of the spatula to cut upon. It has occurred to me that, in a case where excision is preferred, and where the facilities I describe do not exist, the spatulum might be armed on the reverse side with two hooks, when it would perform the double office of uterine tractor and spatula, as necessary. The vagina was simply plugged with Ruspini's styptic. There was scarcely any hemorrhage. The patient recovered speedily and perfectly, and in about two years afterwards conceived and carried a living child to the full period. Her labor was easy and natural; and I had an opportunity of examining her at an interval of several years afterwards, when she was quite well, and the uterus, with the exception of the loss of a portion of the neck, was perfectly sound.

Case III. *Portion of Placenta thrown off in Pregnancy.*—A lady, in the seventh month of her fifth pregnancy, was seized with hemorrhage, ascribed to over-exertion. There were no labor pains. On examination, a portion of the placenta was found protruding through the os uteri. The hemorrhage continued for several days, but not to serious extent, and still there was no labor. At length, fœtid grumous discharges, mixed with a little blood, occurred, attended with sense of downward pressure. The portion of placenta descended lower in the vagina ; its connection with the interior of the os separated; and I removed it with very little assistance. As no increase of hemorrhage occurred from this, I thought it unnecessary to plug the vagina. The hemorrhage and discharge ceased, and the patient went on without any inconvenience, except the precaution of keeping the horizontal position for six weeks longer, when she was delivered of a living boy apparently at or near the full time. The edge of the placenta that remained could not be felt near the os, and the portion that came away consisted of the vascular structure without the reflected membranes. There was no discharge of liquor amnii until the labor set in.

I have already had the honor of calling your attention to some of the more rapidly destructive of puerperal diseases in a paper read for me, in my absence, by your secretary, at your Dublin meeting, under the head of purpuric puerperal fever. It is now my intention to allude briefly to other forms of blood-poisoning, but more especially to puerperal arthritis and puerperal gangrene, premising that, when this disease shows itself, it is usually most rapid and unsparing in its onslaught, and no tissue in the body escapes its ravages.

CASE V. *Puerperal Arthritis—Erosion of Cartilages of Elbow, Hip, and Ankle Joints.*—Kenny, three weeks delivered after a difficult and protracted labor, was awakened from sleep in the night by an acute pain in the left groin. In the morning, she observed a swelling in the middle of the thigh, which at the end of two days had completely engaged the entire limb. The pain became less acute as the swelling increased, but never entirely subsided. Some days subsequently to the swelling of the thigh, she was seized with violent pain in the elbow, but did not perceive any swelling. All these symptoms progressively increased, notwithstanding frequent leeching, stuping, poulticing, opiates, and mercury. She was admitted into the hospital on January 28th, 1829; and, on the 30th, there was an obscure sense of fluctuation over the outer third of the thigh. An incision was made into it, but no pus followed. On February 3d, she had a severe rigor; and on the 4th, she died comatose. A *post mortem* examination was made twelve hours afterwards.

The cellular tissue throughout the entire thigh was filled with gelatinous lymph. An extensive abscess extended from nearly one extremity of the thigh to the other, between the periosteum and muscles. The muscles were pale and flabby, and appeared much softer than natural. About one inch of the upper part of the femoral vein contained pus; its inner tissue was vascular, but did not appear to have lymph upon its surface. The synovial membranes of the hip, knee, and ankle-joints, were filled with puriform matter. The cartilage covering the bones of the hip, appeared healthy; whilst that covering those of the knee and ankle was in part removed by absorption, particularly in the ankle, where scarcely a trace of cartilage could be detected. The uterus was vascular, and inclined towards the left side. The lymphatic glands along the iliac vein were enlarged and vascular. The cartilage was removed altogether from the extremities of the bones forming the right elbow-joint. The viscera appeared healthy.

TAPPED 886 TIMES.—Cann reported to the Academy of Medicine, Paris, 1842, the case of a female with ascites fifteen years, who was tapped 886 times, and was cured. All as yet reported cases must yield to this.—*Clinic.*

